# Identification of proprioceptive thalamocortical tracts in children: comparison of fMRI, MEG, and manual seeding of probabilistic tractography

**DOI:** 10.1093/cercor/bhab444

**Published:** 2022-01-18

**Authors:** Julia Jaatela, Dogu Baran Aydogan, Timo Nurmi, Jaakko Vallinoja, Harri Piitulainen

**Affiliations:** Department of Neuroscience and Biomedical Engineering, Aalto University School of Science, Espoo FI-02150, Finland; Department of Neuroscience and Biomedical Engineering, Aalto University School of Science, Espoo FI-02150, Finland; Department of Psychiatry, Helsinki University Hospital, Helsinki FI-00029, Finland; A. I. Virtanen Institute for Molecular Sciences, University of Eastern Finland, Kuopio FI-70211, Finland; Department of Neuroscience and Biomedical Engineering, Aalto University School of Science, Espoo FI-02150, Finland; Faculty of Sport and Health Sciences, University of Jyväskylä, Jyväskylä FI-40014, Finland; Department of Neuroscience and Biomedical Engineering, Aalto University School of Science, Espoo FI-02150, Finland; Department of Neuroscience and Biomedical Engineering, Aalto University School of Science, Espoo FI-02150, Finland; Faculty of Sport and Health Sciences, University of Jyväskylä, Jyväskylä FI-40014, Finland; Aalto NeuroImaging, Aalto University, Espoo FI-02150, Finland; Pediatric Neurology, New Children’s Hospital, Helsinki University Hospital, Helsinki FI-00029, Finland

**Keywords:** magnetic resonance imaging, magnetoencephalography, multimodal, passive movement, primary sensorimotor cortex

## Abstract

Studying white matter connections with tractography is a promising approach to understand the development of different brain processes, such as proprioception. An emerging method is to use functional brain imaging to select the cortical seed points for tractography, which is considered to improve the functional relevance and validity of the studied connections. However, it is unknown whether different functional seeding methods affect the spatial and microstructural properties of the given white matter connection. Here, we compared functional magnetic resonance imaging, magnetoencephalography, and manual seeding of thalamocortical proprioceptive tracts for finger and ankle joints separately. We showed that all three seeding approaches resulted in robust thalamocortical tracts, even though there were significant differences in localization of the respective proprioceptive seed areas in the sensorimotor cortex, and in the microstructural properties of the obtained tracts. Our study shows that the selected functional or manual seeding approach might cause systematic biases to the studied thalamocortical tracts. This result may indicate that the obtained tracts represent different portions and features of the somatosensory system. Our findings highlight the challenges of studying proprioception in the developing brain and illustrate the need for using multimodal imaging to obtain a comprehensive view of the studied brain process.

## Introduction

Childhood and adolescence are a period of intensive structural and functional development in the brain, which is thought to be related to axonal myelination and synaptic pruning (Blakemore 2012). The development of the proprioceptive system, the sense of body position and movement, is one of those processes that may extend through adolescence (Goble et al. 2005; Cignetti et al. 2013; Marini et al. 2017). Proprioceptive receptors located in muscles, joints and connective tissue provide the central nervous system afferent information about the internal state of the musculoskeletal system (Proske and Gandevia 2012). Any deficits in the processing of the proprioceptive information can have a large effect on both motor control and motor learning. This effect can be seen with normal occurrences of fatigue or injury (Proske and Gandevia 2018), or with pathologies of the sensorimotor system such as cerebral palsy (Yardımcı-Lokmanoğlu et al. 2020), even though the objective evaluation of how the proprioception is affected has proven methodologically challenging (Yardımcı-Lokmanoğlu et al. 2020). Knowledge of the brain processes and especially of the white matter (WM) connections involved in the proprioceptive system, would be highly important to understand the neuroanatomical mechanism of the proprioception and its impairments to develop new treatments. Therefore, there is a significant need for reliable methods to quantify the properties of these crucial connections.

Proprioceptive stimulation activates mainly the primary somatosensory (SI) cortex in the postcentral gyrus and to a lesser extent the primary motor (MI) cortex in the precentral gyrus contralateral to the stimulus (Goldring and Ratcheson 1972). The main pathway for proprioceptive afference to reach SI cortex is through the thalamus, more specifically its ventral posterolateral nucleus (VPL; Gilman 2002; Catani and de Schotten 2012). In addition, to direct proprioceptive input, both SI and MI cortices receive integrated sensorimotor information through widespread and often reciprocal cortico-cortical connections especially from the premotor cortex, supplementary motor area, posterior parietal cortex, and also between the SI and MI cortices (Jones and Porter 1980).

Thalamic WM connections can be studied with diffusion-weighted magnetic resonance imaging (dMRI) and tractography. For example, tractography has been used to indicate the spatial organization of thalamocortical connections on the thalamic surface (Behrens et al. 2003; Johansen-Berg et al. 2005; Traynor et al. 2010) and differences in hemispheric asymmetry, with the left thalamus having significantly higher overall cortical connectivity than the right thalamus (Alkonyi et al. 2011). Thalamocortical sensorimotor tracts have shown to be altered in motor impairments in children, e.g., in cerebral palsy (Hoon Jr et al. 2009; Rose et al. 2011; Papadelis et al. 2014; Kuczynski et al. 2017), and these structural alterations have shown to associate with proprioceptive performance (Fling et al. 2014; Kuczynski et al. 2017).

However, the definition and extraction of a specific white matter tract is not straightforward. All tractography methods require several preset parameters and constraints, most importantly the definition of endpoints of the tracts (i.e., seed and end). In the case of thalamocortical tracts, the respective streamlines are naturally required to originate from the thalamus, which is commonly segmented with an automated atlas. The cortical end of the streamlines is typically restricted to a rather large cortical area such as the postcentral gyrus (Nair et al. 2013; Fling et al. 2014; Papadelis et al. 2014). This coarse endpoint definition, however, disregards the functional fine details within the respective anatomical cortical region, such as the somatotopic organization of different body parts in the SI cortex (Penfield and Jasper 1954). Another commonly used approach is to limit the streamlines to reach manually drawn, spatially more specific regions-of-interest (ROIs) (Sudhyadhom et al. 2013; Jang and Seo 2015; Kuczynski et al. 2017; Tian et al. 2018; Parikh et al. 2019). Manual selection of ROIs requires expertise and is therefore prone to inter-rater variation that may impair their spatial validity and reproducibility. In addition, both manual and atlas-based definitions of WM tracts are unable to address the with-in cortex functional specificity. This can be a major issue especially in the presence of cortical reorganization that is typical for patients with cortical lesions and other malformations, such as in cerebral palsy (Reid et al. 2016).

An emerging approach to overcome the aforementioned issues is to use functional brain imaging to pinpoint the cortical seeds for tractography. This approach, i.e., functional seeding, has been adopted to determine the cortical ROIs using transcranial magnetic stimulation (TMS) (Frey et al. 2012; Weiss et al. 2015; Rosenstock et al. 2017; Sollmann et al. 2020), magnetoencephalography (MEG) (Gaetz et al. 2010; Meng et al. 2010; Papadelis et al. 2018) or functional magnetic resonance imaging (fMRI) (Guye et al. 2003; Gschwind et al. 2012; Bernier et al. 2014; Oechslin et al. 2018). The reliability of functionally seeded tractograms has previously been demonstrated with the corticospinal tract (Smits et al. 2007; Radmanesh et al. 2015; Niu et al. 2016). However, to our knowledge, there has been only one study comparing different functional imaging methods for seeding of tractography. In this study, TMS was evaluated to be more anatomically plausible than fMRI in functional seeding of hand-, foot-, and face-related corticospinal tracts in patients with intracranial tumors (Lucas et al. 2017).

Functional seeding is a promising approach, but the evaluation of using different functional neuroimaging methods is lacking. Here, we aimed to investigate how manual seeding and functional seeding specific to proprioceptive stimulation of the hand and foot affect the thalamocortical tract properties. This is the first time MEG, fMRI, and manual seeding approaches have been examined within the same group of individuals. To our knowledge, this is also the first study to investigate spatial differences of cortical MEG and fMRI responses to proprioceptive stimulation (i.e., to evoked movements) in the same group of individuals. Validation and comparison of these methods in children and adolescents is highly important from the developmental aspect of the proprioceptive and somatosensory systems, especially because comprehensive knowledge is currently lacking in this regard.

The aims of this study were to: 1) compare the localization of manually selected proprioceptive areas, and fMRI-based and MEG-based proprioceptive representations in SI cortex; and 2) study the feasibility of these three approaches in examining proprioceptive thalamocortical tracts in normally developed children and adolescents. It has been previously shown that MEG and fMRI response locations show good congruency in the SI cortex when using tactile stimulation (Kober et al. 2001; Schulz et al. 2004; Zimmermann et al. 2019) or in the MI cortex using active motor tasks (Sanders et al. 1996; Zimmermann et al. 2019). We hypothesized that the MEG and fMRI responses to proprioceptive stimulation would be similarly represented in the SI cortex. Because of the vast connectivity between the thalamus and cortex, we further hypothesized that all approaches would extract robust streamlines, although the spatial location or diffusion properties of the tracts might differ slightly.

## Materials and Methods

### Participants

Thirty-five typically developed children and adolescents (age range 10–18 years) volunteered in this study. We were unable to perform MEG or MRI measurements for 4 participants because of their anxiety towards the measurement situation. From the 31 who were recorded, 12 had to be excluded from further analysis for the following reasons: left-handed (2 participants), on medication (1 participant), or insufficient data quality of fMRI (7 participants), MEG (1 participant) or structural MRI (1 participant). This resulted in a total number of 19 (14 female, age mean ± standard deviation 14.19 ± 2.45 years, age range 10.5–17.7 years) participants in the final analysis. All participants attended MEG and MRI recordings that were carried out either during the same day (14 out of 19) or on two different days with a maximum of 16 days apart (5 out of 19).

The 10-item Edinburgh Handedness Inventory (Oldfield 1971) was used to confirm that all participants were right-handed (test scale: −100 [purely left-handed]–100 [purely right-handed]; mean ± standard deviation: 82.74 ± 18.96; range: 43–100). The participants had no history of neurological disorders or brain injuries.

The study was approved by the Helsinki University hospital ethics committee (HUS/2318/2016) and was in accordance with the recommendations of the Declaration of Helsinki. Informed written consent was obtained from all participants and their guardians before the experiment was conducted.

### MEG Data Acquisition

MEG recordings were performed using a whole-scalp 306-channel MEG system (Elekta Neuromag™, Elekta Oy, Helsinki, Finland) at MEG Core, Aalto NeuroImaging. The recordings were done inside a magnetically shielded room (Imedco AG, Hägendorf, Switzerland). All signals were sampled at 1 kHz using a passband filter of 0.1–330 Hz. Continuous head position was recorded time-locked with the MEG signals using five active head position indicator coils located on the scalp (Fastrak, Polhemus, Colchester, VT, USA). The locations of the coils with respect to anatomical fiducials and scalp surface were determined with an electromagnetic tracker prior to the MEG measurement. Electro-oculogram signal was measured using a pair of electrodes that were placed vertically below and above the lefteye.

### MEG Experiment Protocol

**Figure 1:**
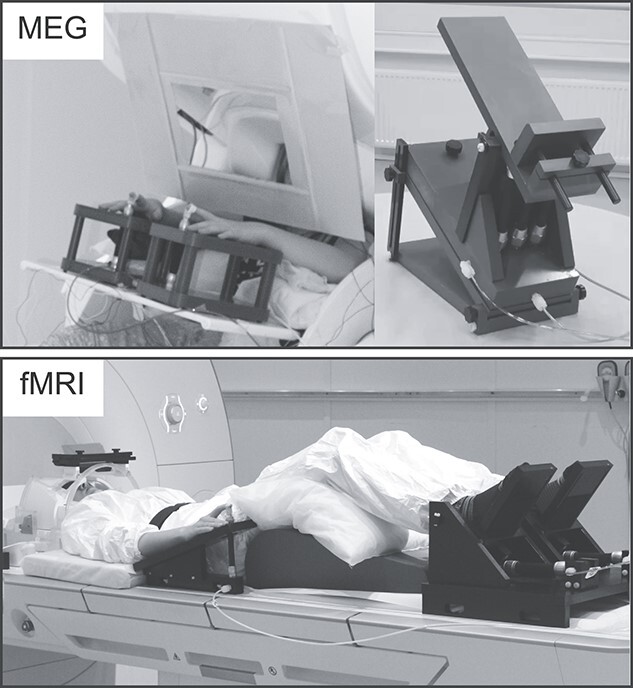
Experimental setup in MEG and fMRI. Proprioceptive stimulation was performed using custom made pneumatic-movement actuators to evoke index finger movements and ankle joint rotations.

Figure 1 presents the MEG setup. Custom-made non-magnetic pneumatic movement actuators (Aalto NeuroImaging, Aalto University, Espoo, Finland) were used to evoke the proprioceptive stimulation of the right and left index finger and ankle joint. Finger extensions of ∼5 mm were evoked when the pressure of the pneumatic muscle (DMSP-10-100 AM-CM, Festo AG & Co., Esslingen, Germany) was quickly reduced from 4 bars to 1 (for a detailed description see Piitulainen et al. 2015). Similarly, ~12° passive dorsiflexions of the ankle joint with peak angular velocity of 26°/s were evoked by pneumatic muscles rotating a footrest when their air pressure was increased from 1 bar to 4 bar (for the details see Piitulainen et al. 2018).

In total, 60 stimuli were delivered for each limb. The order of the stimuli was randomized so that successive stimulations of the same limb and same extremity (upper or lower) were prevented. The inter-stimulus-interval was 4000 ms with a jitter of 250 ms. MEG recording lasted for ∼16 min.

The participants were instructed to sit as relaxed as possible and keep their eyes fixated on an uneventful video that showed slowly moving landscape images. Since the movement actuators caused slight but audible noise, the participants wore earplugs and a constant Brownian noise was played from a panel speaker (Sound Shower, Panphonics, Espoo, Finland) to mask any stimulus-related noises. To minimize tactile stimulation during the evoked movements, a layer of surgical tape was used to cover the fingertips of the index fingers. A cardboard screen was used to prevent the participants from seeing the passive movements of the hands orfeet.

### MRI Data Acquisition

MRI data was collected using a 3 T MAGNETOM Skyra MR scanner (Siemens Healthcare, Erlangen, Germany) with a 32-channel head coil. MRI measurements were done at the Advanced Magnetic Imaging Centre of Aalto NeuroImaging. The imaging was performed without sedation or medication, while the participants were awake and relaxed.

The imaging protocol consisted of structural, functional and diffusion-weighted sequences. The whole MRI session lasted for ~75 minutes with a short break in the middle. During the scanning, the participants wore a respiration belt around their chest and a pulse oximeter was attached to their left middle or ring finger. The physiological signals were recorded with BIOPAC MP150 system (BIOPAC Systems Inc., Goleta,USA).

The structural images were obtained with a T1-weighted magnetization-prepared rapid-acquisition gradient-echo (MPRAGE) sequence [voxel size = 1 mm^3^; field of view (FOV) = 256 × 256 mm; reconstructed matrix = 256 × 256; slices = 176; repetition time (TR)/echo time (TE) = 2.53 s/3.3 ms; flip angle = 7°]. For functional scans, a standard echo-planar imaging (EPI) with spin-echo sequence was used [voxel size = 3 mm^3^; FOV = 192 × 192 mm; reconstructed matrix = 64 × 64; slices = 44; TR/TE = 2.5 s/30 ms; flip angle = 90°].

Diffusion-weighted images (DWIs) were acquired using a single-shot EPI spin echo pulse sequence [voxel size = 2.5 mm^3^; FOV = 240 × 240 mm; reconstructed matrix = 96 × 96; slices = 70; TR/TE = 8.3 s/81 ms; flip angle = 90°]. For each participant, 64 gradient directions with *b* = 1000 s/mm^2^ and 8 acquisitions with *b* = 0 s/mm^2^ were measured.

### fMRI Experiment Protocol

Figure 1 presents the fMRI setup. The proprioceptive stimuli in the fMRI protocol were otherwise the same as in the MEG protocol, but were continuous and performed in a prone position, using adapted versions of the finger (for details see Nurmi et al. 2018) and ankle joint actuators (Nurmi et al. 2021). Both devices consisted of a plastic frame and an artificial pneumatic muscle and operated similarly to the actuators used in the MEG experiment. The pressure of the pneumatic artificial muscle was varied between 1 to 5 bars. Continuous movements at 3-Hz for index fingers and at 1-Hz for ankle joints were used to obtain the blood-oxygen-level-dependent (BOLD) responses. The used frequencies are in the range of natural physiological movements, and the most optimal frequency for index finger BOLD response has shown to be ~ 3-Hz when using the same stimulator (Nurmi et al. 2018).

The fMRI protocol was divided into two 11 and 22-minute runs. In both runs, a block-design was used, in which a 20-s-stimulation block was always followed by a 20-s-rest block. Each limb was stimulated separately: passive movement of 1) right index finger, 2) left index finger, 3) right ankle, and 4) left ankle. There were in total 12 block lists that each included stimulation of each limb once and the rest block was between the successive stimulation blocks. The order of the limbs was randomized within each block list. The first run included four and the second run the remaining eight block lists (for a schematic illustration of the protocol, see Nurmi et al. 2021).

During the scanning, participants were laying on the scanner table in a head-first-supine position (Fig. 1). A pair of earplugs and additional foam pillows were used for hearing protection. Right and left hand of the participants rested on the movement actuators and the index fingers were fixed to the devices using surgical tape. A layer of surgical tape was also used to cover the fingertips of the index fingers to minimize any tactile stimulation. Participants had their feet resting on the movement actuator secured with elastic straps and their legs were supported by pillows for comfortable position. The participants were instructed to stay as still and relaxed as possible and fixate their eyes on abstract images that changed slowly every 30 s. Images were presented using a projector outside the MRI room, back-projection screen, and mirrors.

### MEG Data Processing

MEG data was first denoised using oversampled temporal projection method (Larson and Taulu 2017) that attenuates sensor-specific noise and artifacts efficiently. Additionally, temporal-signal-space separation (Elekta MaxFilter; Taulu and Simola 2006) was used to correct for head movement and to suppress any external interference. Ocular artifacts and remaining cardiac activity induced artifacts were removed using an independent component analysis method (fastICA; Hyvärinen and Oja 1997) calculated with 30 components. All removed components were verified manually.

MEG data was band-pass filtered between 1 and 40 Hz. Data was then divided to baseline corrected epochs −0.5–0.5 s with respect to the onset of the stimulus. Noise covariance was estimated from the 0.5 s pre-stimulus periods in each epoch. Epochs were averaged to produce one average evoked response per stimulustype.

Cortical reconstruction and volumetric segmentation of the anatomical T1-weighted structural images were performed using the Freesurfer image analysis suite (http://surfer.nmr.mgh.harvard.edu/; Dale et al. 1999; Fischl et al. 2002; Segonne et al. 2007; Fischl 2012). For cortical parcellation we used Desikan-Killiany Atlas (Desikan et al. 2006). MNE software (Gramfort et al. 2014) was then used to create a surface-based source space with 8196 source vertices (4096 for each hemisphere) with less than 5 mm distance between them on average. Each vertex represents a point with three orthogonal dipoles. Then, the MEG forward solutions for the source spaces were created (Gramfort et al. 2013).

Source location estimation was done using sLORETA algorithm, which is a normalized minimum norm estimate that has zero localization error for single dipole sources (Pascual-Marqui 2002). Source orientation was loosely constrained to cortical normal direction (orientation weight parameter 0.2). The peak response location was determined by identifying the peak location at the earliest prominent cortical response component that rose significantly above the baseline activity. For each limb, we selected the single voxels at the peak response locations, and call them *MEG*-*ROI*s from nowon.

### MRI Data Processing

All MRI data were visually checked for any pronounced artifacts. DWI data were first converted to 4D NIFTI files. Motion correction and eddy current corrections were done using FMRIB’s Software Library tools eddy and top-up (FSL version 6.0; Andersson et al. 2003; Smith et al. 2004; Jenkinson et al. 2012). An estimate of the susceptibility-induced off-resonance field was obtained from b = 0 images that were gathered in both posterior–anterior and anterior–posterior phase encoding direction. Any remaining geometric deformation were removed using ExploreDTI software (version 4.8.6; Leemans et al. 2009) where we used T1-image as an undistorted reference modality and allowed non-linear deformations only along the anterior–posterior direction. At this step, the diffusion data were co-registered to anatomical T1 data and interpolated to 1 mm^3^ voxelsize.

The fMRI data were converted to NIFTI format and preprocessed using SPM12 software (Wellcome Department of Imaging Neuroscience, University College London, UK) with a custom script on Matlab (R2016b, Mathworks, Natick, Massachusetts, United States of America). All functional volumes were slice-time-corrected, motion-corrected by realigning to the last functional volume and co-registered to anatomical volume. To remove artifacts caused by respiration or pulsation, we used the Drifter tool (Särkkä et al. 2012) together with recorded pulse and respiration signals. The fMRI data were smoothed with a 6-mm kernel and the signal drift was removed by applying temporal high-pass filters of 334 and 658 s.

Next, a design matrix was constructed with the timing information of the stimulation blocks. The design matrix also contained six movement regressors to remove any remaining movement artifacts. A canonical hemodynamic response function was convolved with the stimulation columns in the design matrix. To analyze the relevance of the signal of each voxel in response to the four different stimuli we used a general linear model (GLM). The obtained beta values were used to construct individual contrast images for each stimulus.

To find relevant activity locations, we used the Marsbar toolbox (MARSeille Boîte À Région d’Intérêt; Marseille, France; version 0.44). The threshold p-value of the contrast image was adjusted so that there was a visible activation in the primary sensorimotor (SMI) cortex of the contralateral hemisphere to the proprioceptive stimulation. We then fixed a sphere of 8 mm radius around the local maximum of this activation area. Then, we took the intersection of the sphere and contrast image activation above the selected threshold. From this intersection, a center-of-mass was selected as the primary activation location. Further, these primary activation locations were projected manually on the WM surface of the individual T1-images, in order to efficiently compare the locations obtained from fMRI and MEG, and to ensure standardized tractography (Guye et al. 2003; Smits et al. 2007; Oechslin et al. 2018). The manually selected single voxels on WM surface are called *fMRI-ROI*s from nowon.

### Manual Regions-of-Interest

For tractography analysis, a set of ROIs was defined based purely on the individual anatomical T1-images. First, Freesurfer image analysis suite was used to segment the left and right thalamus for each participant (Fischl et al. 2002) and these segmentations were verified visually on the color-coded FAmap.

Second, the hand and foot areas were located manually based on the well-known homuncular organization of the SI cortex (Penfield and Jasper 1954) and a *manual-ROI* was selected for the left and right finger and ankle joint in the contralateral hemisphere. The index finger location followed the definition of “hand-knob” landmark (Yousry et al. 1997), and the ankle area was selected to be on the medial wall of the paracentral lobule. Spatial localization in the SI cortex for proprioceptive stimulation have been previously reported for the feet using fMRI (Dobkin et al. 2004; Ciccarelli et al. 2005; Francis et al. 2009) or MEG (Piitulainen et al. 2015), and for fingers using MEG (Alary et al. 2002; Onishi et al. 2013; Piitulainen et al. 2015; Hakonen et al. 2021) or fMRI (Shriver et al. 2013; Lolli et al. 2019). These localizations have been consistent with the SI cortex homunculus (Penfield and Jasper 1954).

Based on these previous results, manual-ROIs were placed on the WM surface and the symmetry of the left and right hemispheres was used to guide the selection. Manual placement of ROIs was done by author JJ and verified by author JV with a good agreement.

### Probabilistic Tractography Analysis

Further processing of diffusion data was done with ExploreDTI software. First, we estimated voxelwise diffusion tensors using robust extraction of kurtosis indices with linear estimation algorithm (REKINDLE; Tax et al. 2015) implemented in the software. This method is able to detect and exclude movement-induced artifacts on the data. Second, we used the compartment model approach (Tran and Shi 2015) to estimate fiber orientation distributions (FOD) that are represented with eight-order spherical harmonics. In contrast to diffusion tensor imaging (DTI), this method is capable of representing complex WM structures, e.g., crossing fiber, it also allows for the separation of intra- and extra-axonal components of the signal.

For fiber tracking, we used the state-of-the-art parallel transport tractography algorithm (PTT) (Aydogan and Shi 2020) that is implemented in Trekker (https://dmritrekker.github.io/). PTT is a probabilistic fiber tracking approach that propagates by forecasting a future segment of the streamline using a geometrically smooth fiber model called the probe. For tracking, we used the following parameters: probeLength = 0.2 mm, minRadiusOfCurvature = 0.4 mm, minFODamp = 0.01, and dataSupportExponent = 0.25, other parameters were set to default. In order to provide anatomical constraints to the tracker, diffusion data was first co-registered to the anatomical T1 image, which allowed us to directly use Freesurfer derived anatomical labels. Firstly, 1 million streamlines were generated using random starting points within the Freesurfer-derived left or right thalamus for tracts in the left or right hemisphere, respectively. The part of streamlines that were inside the thalami were removed using the *dontWriteSegmentsInSeedROI* option of Trekker. Cortical reconstruction was used to exclude fibers crossing the midline of the brain or fibers jumping across any sulcus. Streamlines were then filtered to reach either 1) MEG-ROI, 2) fMRI-ROI, or 3) manual-ROI for the right finger, left finger, right ankle or left ankle. All ROIs were initially the size of 1 mm^3^ and were located on WM surface. To address the possible registration and fusion mismatch, the ROIs were dilated using a spherical kernel of 3 mm.

All analyses on fiber trajectory properties were performed in individual anatomy. Spurious streamlines were removed by thresholding the fiber to bundle coherence measure (Meesters et al. 2016) using Dipy (Garyfallidis et al. 2014). For that, we used the recommended parameters in Dipy documentation. The resultant fiber bundles were visually verified. Then track density images (TDI) (Calamante et al. 2010) were computed using Mrtrix (Tournier et al. 2019). Tract masks were obtained by marking all non-zero voxels in TDI as one. To assess the spatial similarity between the 1) fMRI-ROI, 2) MEG-ROI, and 3) manual-ROI based tracts, we calculated the overlap percentages between each method. This was done by dividing the overlapping volume (number of voxels) by the volume outside the overlap (i.e., overlap (%) = 100 ^*^ [A_overlap_/(A_1_ + A_2_—A_overlap_)], where *A_1_* and *A_2_* are tract volumes of fMRI-, MEG, or manual-ROIs). In order to study the microstructural properties of the tracts, we mapped fractional anisotropy (FA), apparent diffusion coefficient (ADC), and apparent fiber density (AFD; Raffelt et al. 2012) onto the streamlines and then computed their average value.

### Statistical Analysis

Statistical analysis was performed using R statistical software (version 4.0.4). We compared the ROI locations and resulting tract properties between fMRI, MEG and manual pipelines using Kruskal-Wallis H test (Kruskal and Wallis 1952), which is a non-parametric version one-way analysis of variance (ANOVA). It is used to conclude whether the independent samples from two or more groups originate from the same distribution. In the case of statistically significant differences (*P* < 0.05), Conover post hoc test (Conover 1999) was used to determine possible pair-wise differences. Post hoc tests were FDR-corrected (Benjamini and Hochberg 1995) for multiple comparisons. F-test was used to denote possible statistically significant differences between variances of MNI coordinates of the ROIs. Finally, to compare left and right hemispheric differences in the tract parameters derived from the three approaches, the right and left side values were pooled together, and were then compared with Mann–Whitney U-test (Mann and Whitney 1947).

## Results

Localization of proprioceptive response in MEG and fMRI was successful in all the 19 participants with few exceptions; one right finger fMRI location (1 out of 19) and three left ankle MEG locations (3 out of 19) were excluded from further analysis due to deviant spatial locations combined with unsatisfactory data quality. All acceptable fMRI-ROIs and MEG-ROIs located in the SMI cortex contralateral to the passive movement of the limbs. Movement actuators used to stimulate the proprioceptors of fingers and ankles did not cause any visible interference in either MEG orfMRI.

### Spatial Differences in the Cortical Seed Locations

The fMRI-ROIs and MEG-ROIs were located within the contralateral SMI cortices when overlayed with individual T1-images. The majority of the responses to finger stimulation were localized in the postcentral SI cortex (fMRI 32 out of 37 and MEG 25 out of 38) and the rest in the precentral MI cortex. Responses to foot stimulation were localized in the SI cortex (fMRI 22/38 and MEG 6/35), the paracentral area (fMRI 8/38 and MEG 23/35), or the MI cortex (fMRI 8/38 and MEG 6/35). Manual-ROIs were selected so that they were always in the SI cortex (fingers) or in paracentral area (ankles).

**Figure 2:**
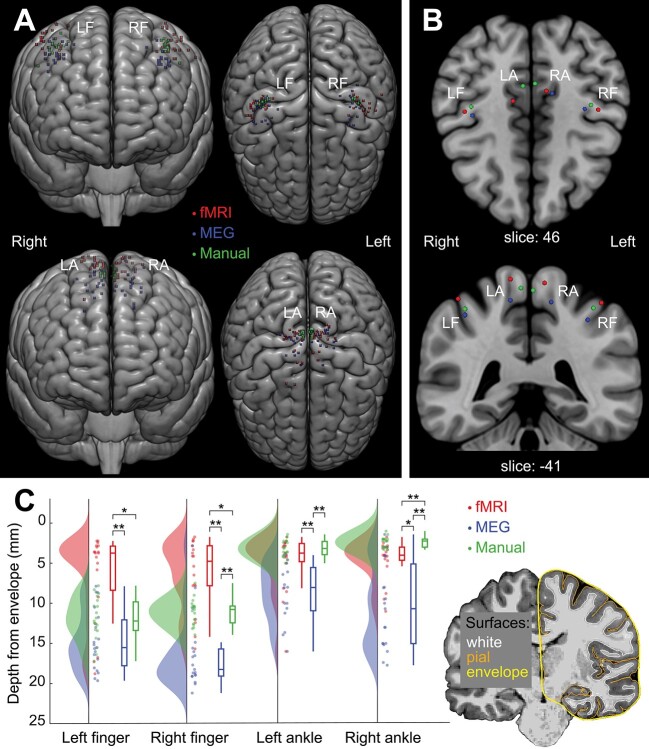
Locations of manually and functionally defined ROIs. (*A*) Individual MNI locations. (*B*) Group mean MNI locations for four limbs. Please note that the locations were transformed to MNI brain only for illustration purposes, and all reported results are derived from the participants’ native space. (*C*) Depth of ROI locations shown with raincloud plots. MEG locations are shown to be situated deeper in the brain (i.e., furthest from the brain envelope) than fMRI and manual locations. Statistically significant differences are shown for each condition with ^*^ = *P* < 0.01 and ^*^^*^ = *P* < 0.001. LF = left finger, RF = right finger, LF = left ankle and RA = right ankle.

Figure 2*A* presents all analyzed fMRI-ROIs, MEG-ROIs, and manual-ROIs in the common MNI brain. Table 1 and Figure 2*B* present the group-mean MNI coordinates. The variances between MNI coordinates differed significantly between the manual and functional proprioceptive locations. MEG-ROIs were more spread out (i.e., variance of MNI coordinates was significantly higher) than manual-ROIs for the right finger (*P* < 0.01) and for both ankles (*P* < 0.01). For ankles, also fMRI-ROIs had a higher variance than manual-ROIs (*P* < 0.01). Variance between MEG and fMRI locations did not differ significantly in either finger or ankle locations.

**Table 1:** Mean MNI coordinates of the ROIs for all limbs

		Left finger	Right finger	Left ankle	Right ankle
fMRI-ROI mean ± SD (mm)	*N*	19	18	19	19
	*x*	42.9 ± 6.2	−43.6 ± 6.5	11.5 ± 4.7	−9.6 ± 5.0
	*y*	−22.8 ± 5.2	−24.1 ± 5.1	−29.2 ± 13.9	−35.6 ± 7.0
	*z*	58.6 ± 7.2	56.3 ± 7.4	71.5 ± 6.1	70.1 ± 5.3
MEG-ROI mean ± SD (mm)	*N*	19	19	16	19
	*x*	38.6 ± 5.4	−35.8 ± 3.9	11.6 ± 4.9	−13.6 ± 5.2
	*y*	−20.5 ± 7.0	−24.5 ± 8.9	−30.3 ± 7.4	−34.6 ± 7.3
	*z*	48.6 ± 5.9	45.8 ± 6.9	58.7 ± 7.8	58.1 ± 9.5
Manual-ROI mean ± SD (mm)	*N*	19	19	19	19
	*x*	39.1 ± 4.5	−39.1 ± 3.0	5.2 ± 1.1	−2.5 ± 0.8
	*y*	−26.1 ± 3.3	−27.3 ± 3.0	−38.9 ± 2.0	−40.5 ± 2.1
	*z*	52.8 ± 5.0	53.5 ± 4.3	67.4 ± 2.4	66.1 ± 2.5

#### Depth of ROI Locations

Figure 2*C* presents the depth of ROI locations for the fMRI, MEG and manual approaches. The depth was determined as the distance from the brain *envelope* (a smoothed outer surface of the pial surface obtained using Freesurfer, see Fig. 2*C*) in the individual anatomy. The depth differed significantly (*P* < 0.001) between the three approaches in all limbs. The ROI locations were deepest for MEG with an average value of 16.0 mm for fingers and 10.0 mm for ankles. For finger locations, the fMRI-ROIs were more superficial (fingers: 6.9 mm, ankles: 5.0 mm) than manual-ROIs (fingers 12.1 mm and ankles 3.1 mm). Pair-wise post hoc testing showed statistically significant differences (*P* < 0.01) between all pairs except left finger MEG–manual comparison (*P* = 0.11) and left ankle joint fMRI–manual comparison (*P* = 0.33).

#### Inter-ROI Distances

**Figure 3:**
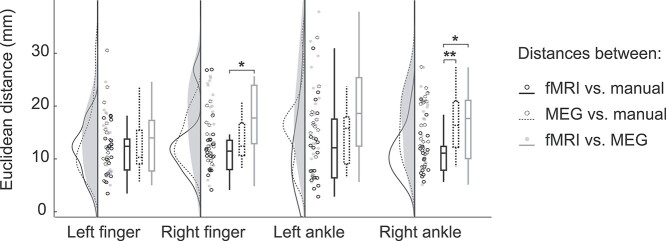
Euclidean distances between the cortical ROIs from fMRI, MEG and manual approaches. The black solid line indicates the distances between fMRI-ROIs and manual-ROIs, the dashed line between MEG-ROIs and manual-ROIs and the gray line between fMRI-ROIs and MEG-ROIs in the participants’ native space. ^*^ = *P* < 0.05, ^*^^*^ = *P* < 0.01.

Figure 3 presents the Euclidean distances between ROI locations of fMRI, MEG, and manual approaches in individual anatomy. The ROIs for the right finger and foot were spatially separated (*P* < 0.05), which was not the case for the left finger or foot (*P* = 0.70 and *P* = 0.08, respectively). For both right finger and ankle joint locations, the fMRI-ROIs were located significantly closer to manual-ROIs than to MEG-ROIs (*P* < 0.05). In addition, right ankle manual-ROIs were located significantly closer to fMRI-ROIs than to MEG-ROIs (*P* < 0.01).

### Thalamocortical Tract Properties

**Figure 4:**
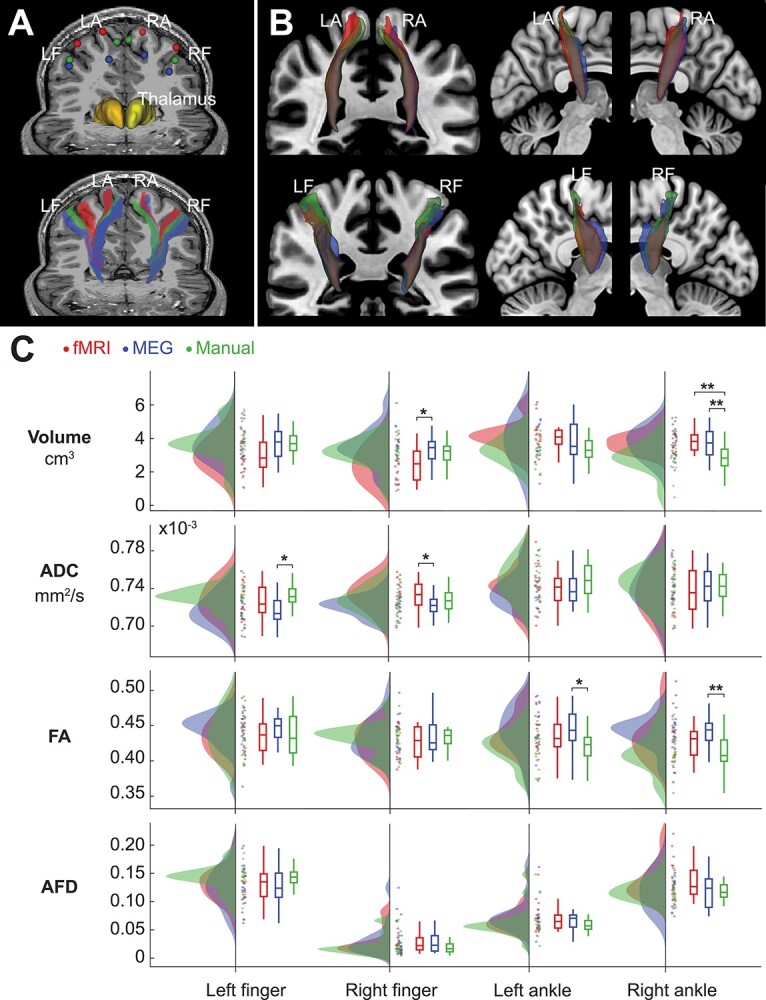
Thalamocortical tracts. (*A*) The upper image shows functional (fMRI and MEG) and manual ROIs (3-mm dilated spherical), and Freesurfer parcellations of left (dark yellow) and right (light yellow) thalamus of a representative participant. The resulting thalamocortical tracts are shown in the lower image. (*B*) The lower image shows group mean tracts for illustrative purposes. Participants’ tract masks were transformed to MNI space and voxels shared by a minimum of 5 participants are visible. (*C*) Tract properties for fMRI, MEG, and manual approaches. Significant differences between the approaches were observed in average tract volume, ADC, and FA, but not in AFD. LF = left finger, RF = right finger, LF = left ankle, RA = right ankle, ^*^ = *P* < 0.05, ^*^^*^ = *P* < 0.01.

Figure 4*A* and *B* shows examples of the extracted thalamocortical tracts. Tractography was performed successfully for all the analyzed 19 participants. After filtering the tracts based on accepted fMRI-ROIs, MEG-ROIs or manual-ROIs, and removing tracts with low streamlines counts (*N*_streamlines_ < 10), the number of participants studied for further analysis was *N* = 15 for left ankle MEG-ROI, *N* = 18 for all right finger tracts and *N* = 19 for therest.

The relative spatial overlap between the thalamocortical tracts derived from fMRI, MEG, and manual approaches were calculated in individual anatomy. The tracts overlapped on average about 1/3 across the four limbs (fMRI vs. MEG 32.4 ± 21.4%, fMRI vs. manual 38.7 ± 21.9%, MEG vs. manual 33.3 ± 21.6%) and there were no statistically significant differences between the overlaps.

Figure 4*C* shows the tract properties of entire tract bundles for the fMRI, MEG and manual approaches. The volume of the right finger and ankle thalamocortical tracts differed significantly between the three ROI types (*P* < 0.05). Post-hoc tests revealed significantly lower volume for manual-ROI based tracts than fMRI-ROIs or MEG-ROIs based tracts in the right ankle tracts (*P* < 0.01), but not in the right finger tracts (*P* > 0.20). In right finger tracts, the fMRI-based tract volumes were lower than MEG-based tract volumes (*P* < 0.05).

Two of the average diffusion properties (ADC and FA) differed between the seeding approaches (Fig. 4*C*). ADC, the measure of mean diffusion, differed between the approaches for both the left and right finger (*P* < 0.05) but not for ankle joints (*P* > 0.21). In the left finger, ADC was significantly lower in the MEG-based than in the manual-based tract (*P* < 0.05). For FA, which describes the degree of diffusion directionality, there were differences in thalamocortical ankle joint tracts (*P* < 0.05) but not in finger tracts (*P* > 0.48). Post hoc analysis indicated a lower degree of anisotropy for manual-based than for MEG-based tracts in both hemispheres (*P* < 0.05). AFD, a relative measure describing the differences in the white matter fiber density, did not differ significantly between the three ROI approaches.

### Hemispheric Asymmetry

**Figure 5:**
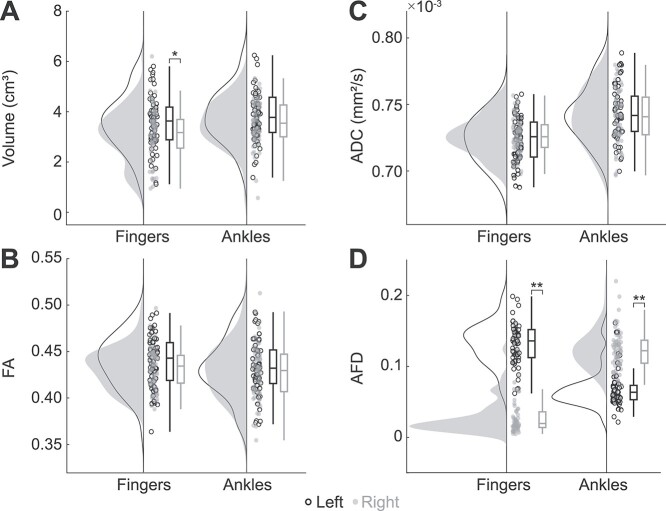
Hemispheric comparison of the thalamocortical tracts. The fMRI, MEG, and manual tract properties were pooled together. (*A*) Tract volume was significantly higher for left finger tracts (black) when compared to right finger tracts (gray). (*B*) FA and (*C*) ADC values were symmetric between the hemispheres. (*D*) AFD values differed between right and left for both fingers and ankles. ^*^ = *P* < 0.05, ^*^^*^ = *P* < 0.001.

Figure 5 presents hemispheric differences for upper and lower limb thalamocortical tracts separately. The tracts from all three seeding approaches were pooled together in the comparison of the two hemispheres. Tract volume was lower for the right finger compared to the left one (*P* < 0.05). In addition, there were statistically significant (*P* < 0.05) hemispheric differences in AFD for both finger and ankle joint thalamocortical tracts. AFD of the right finger tracts was clearly lower compared to left finger tracts (*P* < 0.001). For ankle joint tracts, the difference in fiber density was in the opposite direction, i.e., AFD was higher for right than left ankle tracts (*P* < 0.001). Other tract property estimates, FA and ADC, did not show hemispheric asymmetry.

## Discussion

We compared three different seeding approaches to identify proprioceptive thalamocortical tracts for all four limbs separately in normally developed children and adolescents. The proprioceptors of the hand and foot were stimulated using neuroimaging compatible, in-house developed, fully automated movement actuators to obtain respective functionally relevant fMRI and MEG response locations in the SMI cortex. Thalamocortical tracts were constructed for the functionally (fMRI and MEG) and manually selected seed points, and the tract properties were compared between the seeding methods. Significant differences were found between the seeding approaches both in terms of cortical ROI location and diffusion metrics of the derived tracts. These differences illustrate the challenges of examining specific functionally related WM connections in the human brain such as WM pathways conveying the finger and ankle joint proprioception, and indicate the need for more comprehensive multimodal brain imaging studies.

### The Cortical Seed Locations Differed Spatially between the Seeding Approaches

All three seeding approaches yielded seed locations in the SMI cortex contralateral to the stimulated limb. Thus, our functional proprioceptive locations were overall in good consensus with the locations presented in previous experiments using passive finger movement paradigms in either fMRI (Shriver et al. 2013; Lolli et al. 2019) or in MEG (Alary et al. 2002; Onishi et al. 2013; Piitulainen et al. 2015). Studies using passive ankle stimulation are fewer in number, but similar activation areas can be seen with fMRI (Dobkin et al. 2004; Francis et al. 2009; Choudhri et al. 2015).

Significant spatial differences were found between the seeding approaches in all four limbs. The fMRI activations to the proprioceptive stimulation were more superficial (closer to the brain surface), especially when compared to MEG activations that were deeper in the brain. This clear separation of the functional approaches on the deep-superficial axis is most likely due to methodological differences. MEG is more sensitive to tangential fissural neuronal sources than radial sources at the superficial crest of gyri (Hillebrand and Barnes 2002), whereas fMRI is not restricted by cortical folding. This fundamental limitation of the MEG method likely biases the detected proprioception related cortical activity towards the deeper fissural cortex of the SI and MI cortices.

In addition, MEG and fMRI signals represent partly different portions of the same physiological phenomena. MEG records directly population-level neuronal activity in the cortex, whereas fMRI signal is indirectly related to the neuronal activity through local changes in the cerebral blood flow (hemodynamic response), which might induce some spatial uncertainty to the fMRI-based functional localization (Hall et al. 2014). Taken into account these method-specific limitations, it is not surprising that the MEG and fMRI localizations often differ spatially. In this study, the average Euclidean distances between the MEG and fMRI activations were less than 30 mm, which is in good agreement with previous comparative studies between the two neuroimaging methods (Hall et al. 2014; Klamer et al. 2015). The observed difference in MEG and fMRI localization on the deep-superficial axis is also in line with previous studies of the SMI cortex. For tactile stimulus of fingers, fMRI sources are shown to be located more lateral and inferior to the sources obtained with MEG (Schulz et al. 2004). For the active finger tapping task, fMRI sources are localized more laterally than MEG ones (Klamer et al. 2015).To our knowledge, the spatial differences of fMRI and MEG activation following proprioceptive stimulation have not been previously compared in the same group of individuals.

Although the majority of the obtained functional seed locations were within the SI cortex, some were also localized in the MI cortex, especially among the MEG-based finger locations. Nonhuman primate studies indicate that proprioceptive afference would be primarily processed in SI cortex Brodmann areas 3a and 2, which are located at the bottom curvature of the central sulcus and at the anterior wall of the postcentral gyrus or sulcus (for review see (Jones and Porter 1980; Delhaye et al. 2011). MEG method is especially sensitive to neural activation on the walls of the sulci where the source normal is tangential with respect to the MEG sensor sphere. Therefore, our MEG sources were likely biased more towards area 3a at the bottom curvature of the central sulcus and area 2 at the anterior wall of the postcentral sulcus when compared to fMRI sources. This observation is in line with our previous MEG works where the proprioceptive activation has peaked deep in the central sulcus (Piitulainen et al. 2013). However, the opposite walls of sulci are often hard to separate in MEG due to their proximity, and thus, the response to proprioceptive stimulation of the hand is often detected either at the precentral (MI cortex) or postcentral (SI cortex) wall of the central sulcus, i.e., in the SMI cortex (Piitulainen et al. 2013). Proprioceptive localization in the precentral gyrus is not necessarily a methodological bias, because the MI cortex is also known to receive fast direct proprioceptive input (Goldring and Ratcheson 1972) in a similar fashion as the SI cortex. Furthermore, we restricted the spatial analysis to the spatial peak of the MEG response and center-of-mass of fMRI activation, which ignores the wider spatial distribution seen in MEG and is especially evident in fMRI. The peak location does not necessarily adequately represent the overall cortical activity related to proprioceptive processing. Thus, a multi-seed or adaptable seed shape might be considered for a more optimal definition of the cortical areas for functionally relevant tractography.

The manually defined seed locations, dependent on previous knowledge of proprioceptive cortical locations in adult populations (Francis et al. 2009; Lolli et al. 2019), and tended to be localized in between the functional seeds (MEG and fMRI) for fingers, whereas for ankle joints the manually defined locations were more in consensus with the fMRI locations. Manual finger location was defined to the proximity of the hand-knob, which may not be the optimal location for index finger proprioception in each individual. Recent digit somatotopy studies using ultra-high-field 7 T-fMRI have shown that the index finger cortical representation to tactile stimulation is the most lateral and inferior among the fingers (Stringer et al. 2011; Kolasinski et al. 2016; Sanchez Panchuelo et al. 2018) with an interdigit distance of approximately 7 mm in SI cortex (Stringer et al. 2011). However, the digit somatotopy to proprioceptive stimulation has not been studied. For ankle joints, we chose the mesial wall as the target area based on the previous proprioceptive studies (Dobkin et al. 2004; Francis et al. 2009; Choudhri et al. 2015). However, these studies performed passive ankle dorsiflexion manually (i.e., with help of an assistant) which might affect the accuracy of the reported cortical activation. Our results indicated that fMRI and manual seed locations were in better agreement across all four limbs compared to MEG and manual seed distances; however, this result was significant only for the right ankle. This is a logical observation since the manual seeding approach was primarily based on previous fMRI results.

### Variation in Thalamocortical Tracts Properties between Seeding Approaches

All seeding approaches succeeded in extracting anatomically plausible thalamocortical tracts, but there were differences in the tract volumes and microstructural properties. While we observed significant spatial differences between MEG-ROIs, fMRI-ROIs and manual-ROIs, it is not trivial to anticipate the implications of this on tract properties.

For the manual approach, we found lower tract volume and FA value for proprioceptive ankle tracts but not for the finger tracts. The lower volume and FA values are most likely due to differences in the spatial location of the cortical seeds between the used approaches. Manual seed locations for ankles were placed on the medial wall of the cortex, whereas the functional approaches pinpointed seeds deeper in the gyri. This spatial difference of the seed exposes the manual seeds to be more affected by the gyral bias (Van Essen et al. 2014) that reduces the number of projecting streamlines computed by dMRI-based fiber tractography (Schilling et al. 2018). Lower volumes were also observed in finger tracts that were based on the more superficial fMRI-ROIs, which further supports the effect of gyralbias.

Furthermore, we showed lower ADC values for MEG-based thalamocortical finger tracts compared to the other two seeding approaches. MEG activations were pinpointed deeper in the brain than fMRI or manual locations, which might affect the average diffusivity values. The relationship between ADC and ROI depth from the brain surface requires further investigation. Furthermore, MEG locations were situated more often in the MI cortex, which may impact the ADC values.

In conclusion, the observed differences in the extracted proprioceptive tract properties and their cortical locations indicate that a different portion of the entire thalamocortical connectivity was extracted with the different seeding approaches. It has been shown that distinct thalamic nuclei connect to distinct parts of the cortex (Behrens et al. 2003). Therefore, the point-by-point examination of the thalamocortical connectivity could reveal novel information about the cortical and thalamic structure and could help to understand better how these connections are utilized by the brain.

### Thalamocortical Tracts Showed Hemispheric Asymmetry

Significant hemispheric differences were shown in thalamocortical tract properties. For fingers, the tract volume and average AFD values were higher for the left finger tracts. For the left ankle, the tract AFD was, by contrast, lower than for the right ankle.

Thalamocortical tract volumes can be considered to correlate with connectivity, a measure that has previously been shown to differ between the hemispheres in an adolescent population (Alkonyi et al. 2011). Although the overall connectivity between the thalamus and the cortex was higher for the left hemisphere, the connectivity from the thalamus to the SMI cortex was higher for the right hemisphere (Alkonyi et al. 2011). Our results on proprioceptive thalamocortical tracts show a similar trend: higher tract volume was observed for the left limb afferent tracts, i.e., from the thalamus to the right SMI cortex.

Significantly higher radial diffusivity of right hemisphere thalamocortical proprioception related tracts have been observed in multiple sclerosis patients and healthy controls (Fling et al. 2014). An increase in radial diffusivity results in a decrease in AFD, i.e., lower fiber density (Raffelt et al. 2012). Thus, our observation of the lower AFD for the left ankle thalamocortical tract in the right hemisphere is in line with the observations by (Fling et al. 2014). In their study, all thalamocortical tracts projecting to the SI cortex were examined together, whereas we examined upper and lower extremities separately. Surprisingly, the hemispheric difference in AFD was opposite in upper than lower limbs, which highlights the need for more detailed studies of the specific thalamocortical tracts.

To the best of our knowledge, there is no previous study that showed asymmetry of thalamocortical tracts using AFD measure, which is a relatively new measure for high angular resolution diffusion-weighted images. Therefore, future studies are needed to confirm our results with better quality diffusion images and on a larger sample of adolescents and adults. In addition, the along-tract analysis could reveal more specific information about the functionally relevant thalamocortical tracts, especially at the proximity of the cortex.

### Limitations

#### Children and Adolescents

Examination of the developing brain is more vulnerable to movement artifacts compared to adult studies. Especially for young children, it can be difficult to stay still for prolonged periods in MRI and with a lesser degree in MEG. Although the participants with clear head movements were excluded from the current analysis, the head movement could still partly affect primarily our fMRI results. Another limitation when studying children is the use of atlases and pipelines that are developed for the adult brain. To overcome this issue, all automated processes were verified visually. The sample size of the current experiment was limited (*n* = 19), and the majority of our participants were female (14 out of 19). Thus, the conclusion made in this study may require future investigation with larger sample size, and the potential effect of the gender should be investigated.

#### Functional Imaging

The comparison of functional responses in fMRI and MEG was limited by the differences in the presented proprioceptive stimuli. In MEG, the cortical response was measured after single elicited passive movements in a seated position, whereas in fMRI we used continuous movement in a supine position. Despite these differences, we believe that the used proprioceptive stimulation should result in similar primary response locations in the cortex.

We used precise and reproducible (Piitulainen et al. 2020) neuroimaging compatible movement actuators. Passive movement of the finger or ankle joints inevitably activates some cutaneous mechanoreceptors (e.g., through a stretch of the skin) that can be functionally considered as part of the proprioception and thus is not considered as a major limitation.

Previous studies using passive movement have shown fMRI activation to be somewhat widespread, covering a significant part of the SMI cortex (Francis et al. 2009; Lolli et al. 2019). In this study, some participants had several plausible activation areas in the SMI cortex, of which we manually selected the one to best reflect SI activation. The choice to use center-of-mass as a representative location for fMRI activation further simplifies the activated cortical area to a single point and disregards the variation in activation strength within this area. Moreover, if the fMRI activation has complex geometry and/or large size, the center-of-mass point might not be a particularly representative seed of that region of activation. However, we believe that this method is more robust than using e.g., the peak activation that might lead to larger errors.

For MEG localization, we used sLORETA, which offers zero dipole location error for a single source. However, in presence of multiple sources, the ideal assumptions do not hold and sLORETA can create ghost sources or false maxima (Hauk et al. 2011). We used the first peak of cortical activity to determine the source location but there can still be multiple sources active confusing the source locations.

#### Tractography

Reconstruction of the WM connections from dMRI data is a highly challenging task, mainly owing to the complexity of WM structure with crossing fibers and noise in images that are also typically acquired at low resolutions (Rheault et al. 2020). Overall, it is widely accepted that these challenges lead dMRI-based tractograms to contain large amounts of false positives (Schilling et al. 2019) and false negatives (Aydogan et al. 2018). To diminish the effect of these limitations, we used a multi-compartment based approach to model the diffusion signal, which can represent WM structures with FODs (Tran and Shi 2015). Using fixed values for the diffusivities of compartments, this approach can separate intra- and extra-axonal compartments that enable the use of AFD measure in our single shelled data. Additionally, we used a state-of-the-art probabilistic parallel transport tractography algorithm that reduces noise related propagation errors and generates smooth, gradually changing fiber bundles (Aydogan and Shi 2020).

## Conclusion

Our results highlight the challenges of studying the functional anatomy of the developing proprioceptive system in the human brain. We showed that there are significant differences in fMRI, MEG, and manual localization of proprioceptive areas in the SMI cortex, which affected the microstructural properties of the obtained thalamocortical tract bundles. The functional seeding in tractography is thought to enable a more robust examination of functionally relevant tracts, but we indicated that the neuroimaging modality may significantly affect the results and conclusions drawn. Thus, multimodal neuroimaging seeding approaches would be beneficial for future tractography studies on proprioception or any other sensory modalities.

## Author Contributions

**Jaatela J.** did conceptualization, methodology, formal analysis, investigation, data curation, writing—original draft, visualization, project administration**; Aydogan D. B.** did methodology, formal analysis, resources, writing—review & editing**; Vallinoja J.** did methodology, formal analysis, investigation, data curation, writing—review & editing; **Nurmi T.** did methodology, formal analysis, investigation, data curation, writing—review & editing; **Piitulainen H.** did conceptualization, methodology, resources, resources, writing—review & editing, supervision, project administration, funding acquisition.

## Notes

We thank Helge Kainulainen from his technical support in building the pneumatic system at Aalto NeuroImaging, Aalto University, Espoo, Finland. The calculations presented above were performed using computer resources within the Aalto University School of Science “Science-IT” project. *Conflict of Interest:* None declared.
